# γ-secretase inhibitor I inhibits neuroblastoma cells, with NOTCH and the proteasome among its targets

**DOI:** 10.18632/oncotarget.11715

**Published:** 2016-08-30

**Authors:** Carmen Dorneburg, Annika V. Goß, Matthias Fischer, Frederik Roels, Thomas F.E. Barth, Frank Berthold, Roland Kappler, Franz Oswald, Jens T. Siveke, Jan J. Molenaar, Klaus-Michael Debatin, Christian Beltinger

**Affiliations:** ^1^ Department of Pediatrics and Adolescent Medicine, University Medical Center Ulm, Ulm, Germany; ^2^ Children's Hospital, Department of Pediatric Oncology and Hematology, University of Cologne, Cologne, Germany; ^3^ Department of Pathology, University Medical Center Ulm, Ulm, Germany; ^4^ Department of Pediatric Surgery, Dr. von Hauner Children's Hospital, Ludwig-Maximilians-University, Munich, Germany; ^5^ Department of Internal Medicine I, University Medical Center Ulm, Ulm, Germany; ^6^ Department of Internal Medicine, University Hospital Essen, Essen, Germany; ^7^ Department of Oncogenomics, Academic Medical Center, Amsterdam, The Netherlands

**Keywords:** γ-secretase inhibitor, NOTCH, neuroblastoma, preclinical, *in vivo*

## Abstract

As high-risk neuroblastoma (NB) has a poor prognosis, new therapeutic modalities are needed. We therefore investigated the susceptibility of NB cells to γ-secretase inhibitor I (GSI-I). NOTCH signaling activity, the cellular effects of GSI-I and its mechanisms of cytotoxicity were evaluated in NB cells *in vitro* and *in vivo*. The results show that NOTCH signaling is relevant for human NB cells. Of the GSIs screened *in vitro* GSI-I was the most effective inhibitor of NB cells. Both *MYCN-*amplified and non-amplified NB cells were susceptible to GSI-I. Among the targets of GSI-I in NB cells were NOTCH and the proteasome. GSI-I caused G2/M arrest that was enhanced by acute activation of MYCN and led to mitotic dysfunction. GSI-I also induced proapoptotic NOXA. Survival of mice bearing an *MYCN* non-amplified orthotopic patient-derived NB xenograft was significantly prolonged by systemic GSI-I, associated with mitotic catastrophe and reduced angiogenesis, and without evidence of intestinal toxicity. In conclusion, the activity of GSI-I on multiple targets in NB cells and the lack of gastrointestinal toxicity in mice are advantageous and merit further investigations of GSI-I in NB.

## INTRODUCTION

Neuroblastoma (NB) is the most common extracranial solid tumor of childhood [1, 2]. As the prognosis of children with high-risk NB remains poor, novel therapeutic approaches are needed.

Embryonic, undifferentiated tumors, such as NB, are characterized by constitutive activation of developmental signaling pathways. NOTCH is a developmental pathway determining the fate of neural crest stem cells, the cells of origin of NB [3, 4]. The molecular mechanisms of the NOTCH pathway have been elucidated in great detail (reviewed in [5]). Briefly, cell-bound NOTCH ligands bind to cell surface NOTCH receptors. NOTCH receptors then undergo an extracellular cleavage that generates the NOTCH-EXT fragment. Subsequently, this fragment is cleaved by γ-secretase to generate the intracellular domain of NOTCH (NICD). NICD translocates into the nucleus and binds to the transcription factor RBPJ (recombination signal binding protein, suppressor of hairless). Subsequently, NOTCH target genes such as *DTX1*, *NRARP* and others are induced depending on cell type.

There is emerging, albeit contradictory evidence that NOTCH is involved in established NB. NOTCH receptors are expressed in NB [6–9]. Little is known about the expression of NOTCH ligands in NB [10]. Expression of NOTCH target genes at high levels has been found by some [7–11] and at low levels by others [6]. The cellular effects of NOTCH signaling in NB appear to depend on the triggers, level and duration of NOTCH activation. Thus, triggering NOTCH signaling by recombinant JAGGED1 led to growth arrest [9], while a JAGGED1 peptide enhanced proliferation [12]. Transfection of NOTCH1-3 intracellular domains and HES1 killed NB cells [9], as did increased expression of HES1 by other means [7, 11], whereas hypoxia-associated upregulation of NOTCH1 was linked to an immature neural crest cell-like phenotype [13]. While constitutive NOTCH activation kept NB cells in an undifferentiated state, transient activation induced their differentiation [8, 11]. Finally, increased NOTCH1 protein has been correlated with poor prognosis of NB [6], others, however, found no evidence of cleaved NOTCH in NB [9]. There is evidence that co-expression of NOTCH receptor and ligand in the same cell inhibits the NOTCH receptor (“cis-inhibition”) [14]. This possibility, and the contradictory findings of the role of NOTCH signaling in NB highlight the complexity of delineating NOTCH signaling in NB cells.

Among other options to block Notch signaling, the macromolecular γ-secretase complex is a promising therapeutic target in cancers with active NOTCH [15]. Several small molecule γ-secretase inhibitors (GSIs) have been developed and have entered clinical trials. These compounds inhibit γ-secretases that cleave NOTCH and additional proteins [16–20], inhibit the proteasome and can elicit endoplasmic reticulum stress [21–26]. GSI-I has been shown to inhibit gastric cancer xenografts in mice after systemic administration [27]. Little is known about the efficacy of the various small molecule GSIs in NB [6, 12].

The ubiquitin-proteasome pathway is a major mechanism in intracellular protein turnover and its concerted action is necessary for many cellular processes [28]. The proteasome is a therapeutic target for cancers, including NB, and proteasome inhibitors have been investigated for therapeutic efficacy for more than a decade. However, proteasome inhibitors like bortezomib show low activity when used as monotherapy for solid tumors [29, 30].

Here, we provide evidence that GSI-I is the most effective of the γ-secretase inhibitors and acts on at least two therapeutic targets in NB, NOTCH signaling and the proteasome, leading in concert to cell cycle arrest, mitotic catastrophe and inhibition of NB cell growth.

## RESULTS

### NOTCH signaling is active in human NB

**Figure 1:**
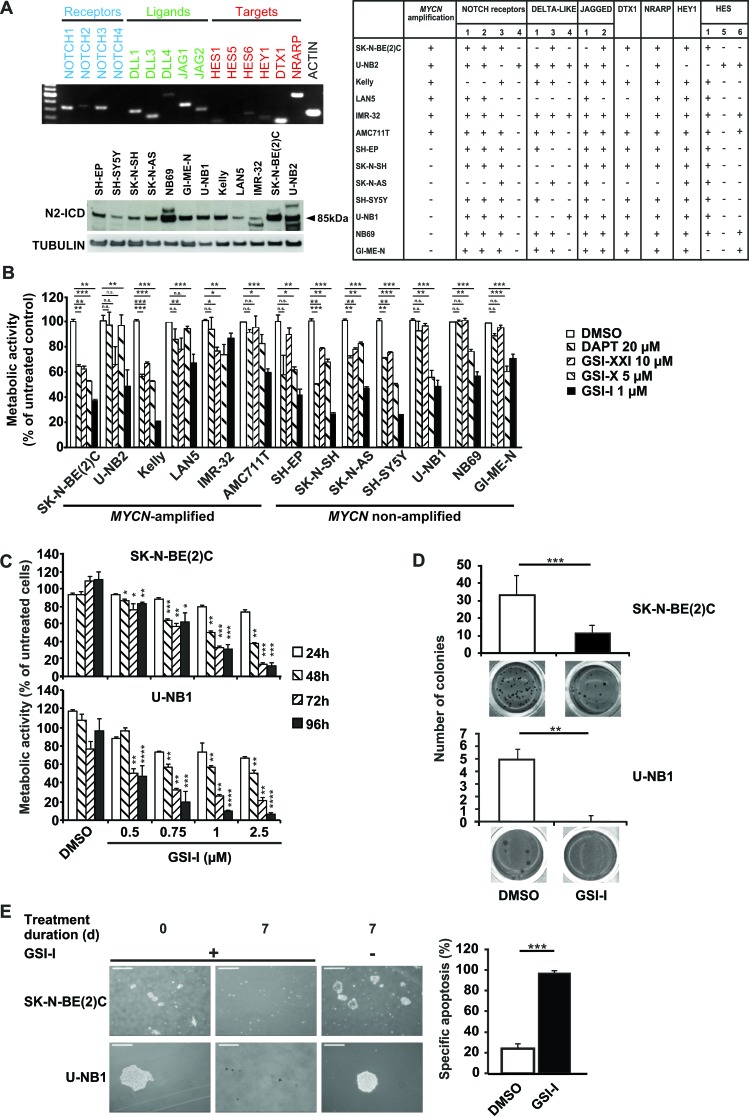
NOTCH signaling is active in human NB cells and inhibition of γ-secretase *in vitro* decreases malignant attributes of NB **A**. All NB cell lines and primary low-passage cultures investigated express at least one NOTCH receptor and one NOTCH ligand leading to activation of NOTCH target genes. Cells were subjected to semi-quantitative RT-PCR for NOTCH receptors (blue), ligands (green) and targets (red) (upper panel). Shown is one representative of three independent experiments with IMR-32. The table summarizes the results of NB cell lines and short-term cultures. Expression is denoted by “+”, lack of expression by “-”. Genomic amplification of *MYCN*, as derived from published data, is marked by “+”, lack of amplification by “-“. NB cells were investigated by Western blot analysis of N2-ICD, tubulin was used as loading control (lower panel). **B**. GSI-I is the most effective GSI against NB cells. 1 × 10^4^ NB cells in 96-wells were treated with different GSIs for 48 h and metabolic activity was assessed by MTT assay. Minimal effective doses determined in previous experiments were used. Results were calculated relative to DMSO controls. **C**. GSI-I decreases metabolic activity in a time- and dose-dependent manner. 1.5 × 10^4^ NB cells were seeded in quadruplets in 96-well plates and treated with the indicated GSI-I concentrations. Metabolic activity was assessed by MTT assay. Results are compared to DMSO controls. **D**. GSI-I markedly decreases anchorage-independent growth. NB cells were seeded at clonal density into 24-well plates in soft agar and were treated with 1 μM (U-NB1) or 1.5 μM GSI-I (SK-N-BE(2)C). Colonies were stained with 3-(4,5-dimethylthiazol-2-yl)-2,5-diphenyltetrazolium bromide and counted in 12 wells per condition. **E**. NB spheres are targeted by GSI-I. SK-N-BE(2)C and U-NB1 cells were seeded in clonal density using non-adherent plastic and tumor sphere medium. GSI-I at 1 μM was applied when spheres became visible. Integrity of spheres was determined by light microscopy (left). Bars represent 250 μm (upper panel) and 60 μm (lower panel). Specific apoptosis was determined by FACS analysis (right). Means and SD are shown in B-E. Statistical analysis was performed using the unpaired t-test. *p<0.05, **p<0.01, ***p<0.001. Experiments in A-E were repeated at least three times in triplicates.

Primary short-term cultures were shown by immunohistochemistry and FISH to be *bona fide* NB cells without lymphocyte contamination (Supplementary Figs. S1 and S2). Using these and other authenticated NB cells, expression of NOTCH receptors and ligands, and target gene activation was investigated. All NB cell lines and cultures expressed at least one of the NOTCH receptors and ligands, leading to induction of NOTCH target genes (Figure 1A, upper panel and table). To confirm activation of NOTCH, the presence of cleaved NOTCH1 (N1-ICD) and NOTCH2 (N2-ICD) was determined. While N1-ICD was detectable at low levels in some NB cell lines and cultures (Supplementary Figure S3). N2-ICD was clearly present in all cell lines and cultures (Figure 1A, lower panel). These data confirm that NOTCH is active in human NB.

### γ-Secretase inhibitor I decreases malignant attributes of NB *in vitro*

We then asked whether NOTCH is a therapeutic target in NB and investigated blocking NOTCH by inhibiting γ-secretase, the common switch in NOTCH activation. We first screened the small-molecule γ-secretase inhibitors GSI-I, GSI-X, GSI-XXI and DAPT in a panel of NB cell lines and short-term cultures for their minimal effective dose using dose-response curves (data not shown). GSI-I was the most potent inhibitor (Figure 1B), while GSI-X, GSI-XXI and DAPT were less effective, even when used at 5-fold higher concentrations than GSI-I. Thus, GSI-I was used throughout the study. For reasons of feasibility we decided to concentrate on the paradigmatic *MYCN*-amplified SK-N-BE(2)C cell line and the non-amplified low-passage culture U-NB1 for most of the subsequent studies. Thus, caution must be exerted in extrapolating the results to all NB cells. Both SK-N-BE(2)C and U-NB1 cells were killed in a time- and dose-dependent manner by GSI-I (Figure 1C). At a concentration of 1 μM GSI-I markedly inhibited metabolic activity of most NB cell lines and of all primary short-term NB cultures investigated (Figure 1C and Supplementary Figure S4). Next, we examined the effect of GSI-I on clonogenicity. GSI-I dramatically inhibited clonogenicity in all 12 NB cell lines and cultures tested (Figure 1D and Supplementary Figure S5). To test whether GSI-I impacts on NB spheres that may be enriched with NB initiating cells, spheres of SK-N-BE(2)C and U-NB1 cells were treated with GSI-I. Spheres disintegrated into single cells, associated with massive apoptosis (Figure 1E). Taken together, all NB cells were sensitive to GSI-I, as GSI-I markedly decreased viability, clonogenicity and tumor sphere integrity.

### GSI-I targets γ-secretase and inhibits NOTCH signaling in NB cells

**Figure 2:**
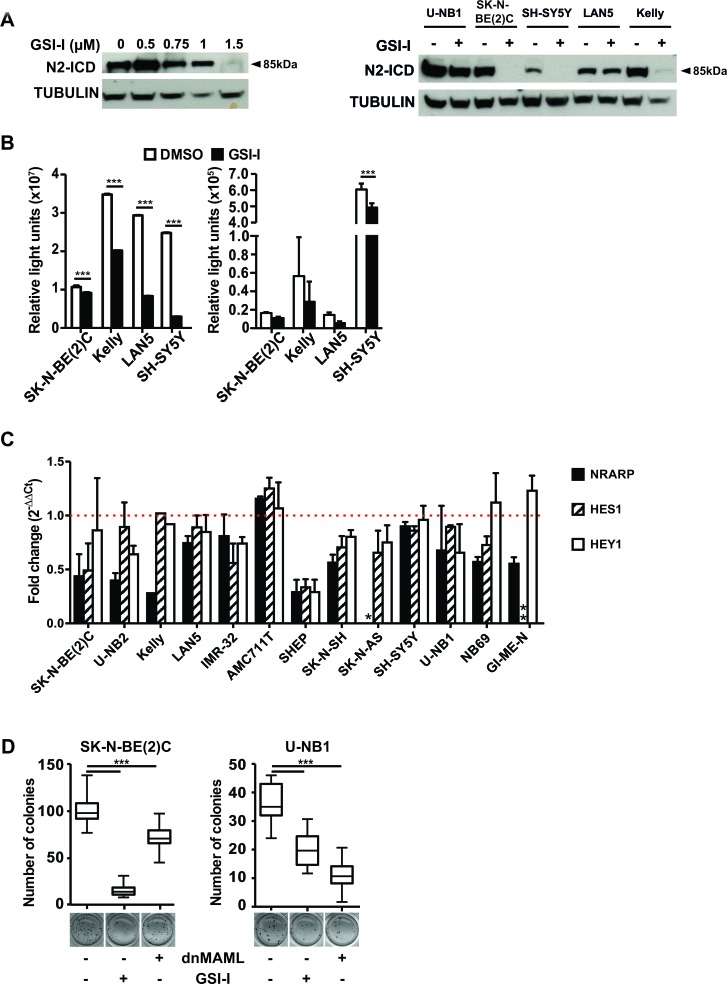
GSI-I targets γ-secretase and inhibits NOTCH signaling in NB cells **A**. GSI-I inhibits γ-secretase-mediated cleavage of NOTCH2 depending on dose. U-NB1 cells were treated with GSI-I at increasing concentrations for 48 h (left panel). Representative NB cell lines were treated with GSI-I at 1 μM for 48 h (right panel). N2-ICD levels were determined by Western blot analysis. Tubulin was used as loading control. **B**. GSI-I reduces induced and endogenous NOTCH signaling. NB cells were co-transfected with mNOTCH1-ΔE and the NOTCH reporter construct RBP-Jκ-luc (left), or were transfected with the reporter construct only (right). 24 h after GSI-I treatment luciferase-activity was measured. Results are depicted as luciferase activity in relative light units (RLU). Shown are means and SD of one out of three independent experiments. P values were calculated using the two-way ANOVA test; ***p<0.001. **C**. GSI-I decreases NOTCH target gene expression. NB cells were treated with GSI-I for 72h. Expression of the NOTCH target genes *NRARP*, *HES1* and *HEY1* was measured by qRT-PCR, normalized to expression of *ACTIN* and is depicted relative to DMSO-treated control samples. The dotted horizontal line (red) indicates the level of NOTCH target gene expression of the DMSO-treated control samples (set as one-fold change). * SK-N-AS cells do not express *NRARP* and ** GI-ME-N cells do not express *HES1*. Means and SD of three independent experiments are shown. **D**. Both GSI-I and genetic NOTCH inhibition reduce clonogenicity. SK-N-BE(2)C and U-NB1 cells were transiently transfected with either dnMAML-GFP (“dnMAML +”) or empty vector control expressing only GFP (“dnMAML –“). Cells sorted above the 50^th^ percentile for GFP were seeded in clonal density (1000 cells/well) in soft agar. Cells were then treated with vehicle only (“GSI-I –“) or with 1 μM (U-NB1) or 1.5 μM (SK-N-BE(2)C) GSI-I (“GSI-I +”). Colonies were stained and counted. P values were calculated using the unpaired t-test; ***p<0.001. Experiments were repeated three times, with similar results.

To assess whether GSI-I targets γ-secretase and inhibits NOTCH signaling in NB cells, we determined the efficacy of GSI-I on γ-secretase-mediated cleavage of NOTCH receptors 1 and 2. GSI-I inhibited cleavage of NOTCH1 in those NB cell lines and cultures were N1-ICD was detectable (Supplementary Figure S3). GSI-I-mediated inhibition of NOTCH2 was much more evident. It occurred in a dose-dependent fashion (Figure 2A, left panel) and in the majority of NB cell lines and cultures investigated (Figure 2A, left and right panels). This shows that GSI-I targets γ-secretase. When mNOTCH1-ΔE, a dominant active form of NOTCH1 constitutively cleaved by γ-secretase, was overexpressed in NB cells, it was clearly inhibited by GSI-I, as detected by a concomitantly transfected NOTCH reporter construct (Figure 2B, left). However, little impact of GSI-I on endogenous NOTCH activity was detectable with this assay (Figure 2B, right). Transcripts of the NOTCH target genes *NRARP*, *HES1* and *HEY1* decreased upon GSI-I, in a heterogeneous fashion (Figure 2C). We conclude that most of these data are consistent with GSI-I I targeting γ-secretase and inhibiting NOTCH signaling in NB cells.

Next, we compared the impact of NOTCH inhibition on clonogenicity by GSI-I with specific, non-pharmacological NOTCH inhibition. GSI-I nearly abrogated and significantly reduced clonogenicity of SK-N-BE(2)C and U-NB1 cells, respectively (Figure 2D). NB cells transfected with dominant-negative mastermind-like protein (dnMAML-GFP), blocked the NOTCH transcriptional activation complex, as proven by decreased *DTX1* expression (Supplementary Figure S6) and reduced clonogenicity of NB cells, more so in U-NB1 than in SK-N-BE(2)C cells (Figure 2D). This differential sensitivity is in line with the modest NOTCH receptor activity in the SK-N-BE(2)C cells (Figure 2B). These data, together with the data showing inhibition of NOTCH signaling by GSI-I, strongly suggest that inhibition of NOTCH signaling contributes to the efficacy of GSI-I in some NB cell lines and less so in others.

### GSI-I inhibits the proteasome in NB cells

**Figure 3:**
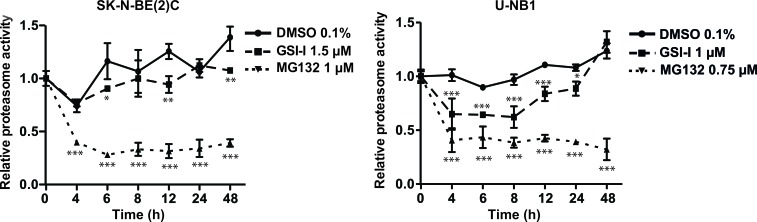
GSI-I inhibits the proteasome in NB cells NB cells were treated in triplicates with either GSI-I or MG132. Proteasome activity was measured using a fluorogenic AMC substrate assay. Means and standard deviations of three independent experiments are shown. P values were calculated using the two-way ANOVA test; *p<0.05, **p<0.01, ***p<0.001.

Given the structural similarity of GSI-I with proteasome inhibitors, we compared the impact of GSI-I on the proteasome of NB cells with that of MG132, a *bona fide* proteasome inhibitor. Proteasome inhibition of GSI-I in SK-N-BE(2)C cells was modest compared to the strong proteasome inhibitor MG132, while proteasome inhibition of U-NB1 was more pronounced (Figure 3). Inhibition was persistent in SK-N-BE(2)C and transitory in U-NB1 cells.

### GSI-I activates the G2/M checkpoint, causes mitotic dysfunction and induces apoptosis

**Figure 4:**
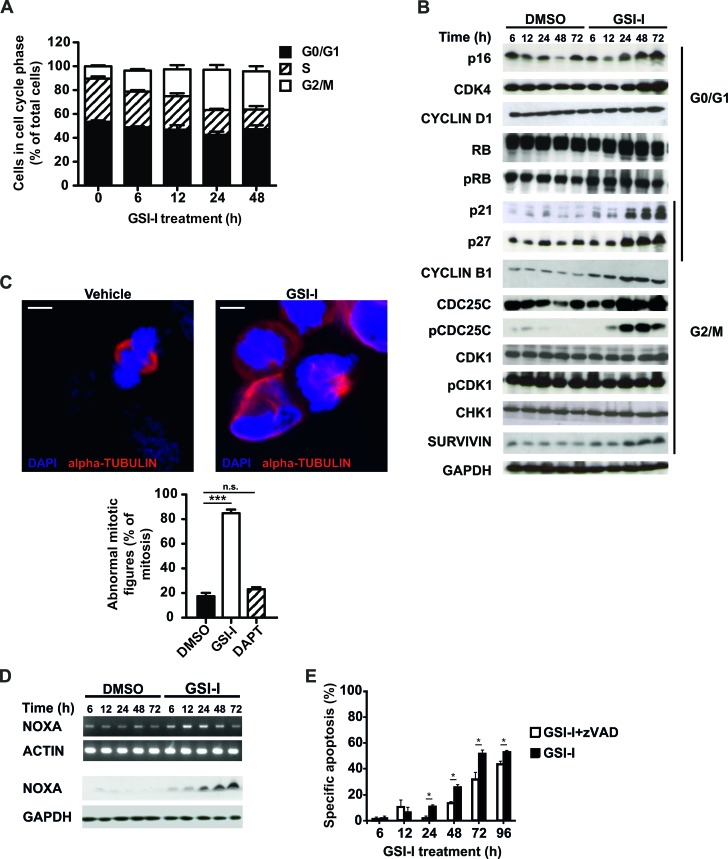
GSI-I activates the G2/M checkpoint and induces mitotic dysfunction in SK-N-BE(2)C NB cells **A**. GSI-I induces G2/M cell cycle arrest. SK-N-BE(2)C cells were treated with 1.5 μM GSI-I for the indicated times. Cells in G1, S and G2/M phases were determined by FACS analysis. Means and SD were calculated from three independent experiments. **B**. GSI-I-induced cell cycle arrest is p53-independent and associated with increased p16, CDK4, p21, p27, CYCLIN B1, CDC25C, pCDC25C and SURVIVIN. Protein lysates of SK-N-BE(2)C cells treated with 1.5 μM GSI-I were subjected to Western blot analysis. Experiments were repeated three times, with similar results. **C**. GSI-I induces mitotic dysfunction. SK-N-BE(2)C cells were treated on cover slips with 1.5 μM GSI-I or DAPT for 24 h. Formalin-fixed samples were stained for α-tubulin (red) and DNA (blue). The graph shows the quantification of aberrant mitotic figures and is presented as percent of total mitotic figures. Bars equal 5 μm. Experiments were repeated three times, with similar results. Shown are means and SD from three independent experiments and p-values were calculated using the t-test. *** p<0.001; n.s. not significant. **D**. GSI-I increases NOXA mRNA and protein. NOXA in GSI-I-treated SK-N-BE(2)C cells was determined by semi-quantitative RT-PCR (upper panels) and Western blot (lower panels). Experiments were repeated three times, with similar results. **E**. GSI-I-induced apoptosis is partially dependent on caspases. SK-N-BE(2)C cells were treated with 1.5 μM GSI-I in the presence or absence of zVAD.fmk. Hypodiploid propidium iodide-stained nuclei were determined by FACS analysis. Means and SD were calculated from three independent experiments and p-values were determined using the unpaired t-test; *p<0.05.

**Figure 5:**
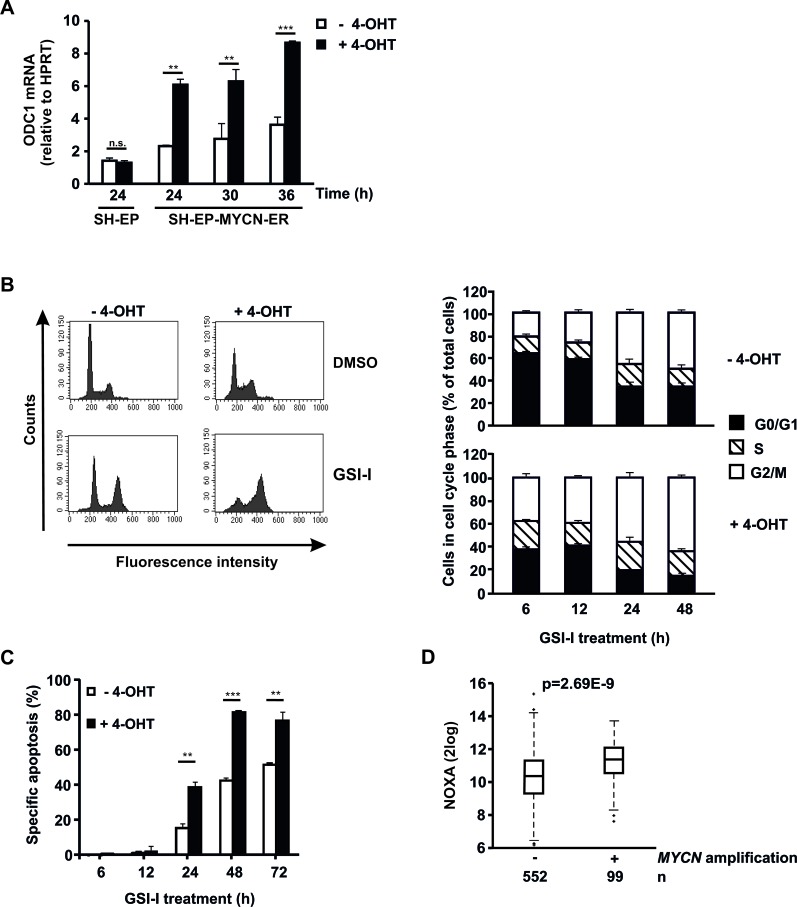
Acute activation of MYCN sensitizes NB cells to GSI-I **A**. MYCN translocation induces *ODC1* expression. Parental SH-EP and SH-EP-MYCN-ER cells were treated with 4-OHT. qRT-PCR of *ODC1* was performed. mRNA expression was calculated relative to *HPRT*. Means and SD were calculated from independent qRT-PCR runs and p-values were determined using the two-way ANOVA test; **p<0.01, ***p<0.001. **B**. MYCN forces NB cells into S phase and enhances GSI-I-induced G2/M block. SH-EP-MYCN-ER cells were incubated with 4-OHT for 24 h and subsequently treated with 1.5 μM GSI-I. PI-stained cells were analyzed by flow cytometry. Histograms for the 48 h time point are shown on the left and quantification of cells being in G0/G1, S and G2/M phases on the right. Means and SD were calculated from three independent experiments. **C**. MYCN enhances GSI-I-induced apoptosis in NB cells. SH-EP-MYCN-ER cells were treated as described in (B). Hypodiploid nuclei were determined by FACS analysis. Experiments were repeated at least three times, with similar results. For statistical analysis the unpaired t-test was used; **p<0.01, ***p<0.001. **D**. *MYCN* amplification is associated with increased *NOXA* transcripts in patient NB. Transcript levels of *NOXA* were determined in 651 patient NB by gene expression microarray analysis. Statistical analysis was performed using the Wilcoxon Rank Sum test.

To investigate effector mechanisms of GSI-I-induced cytotoxicity we performed cell cycle analysis in *MYCN*-amplified SK-N-BE(2)C and *MYCN* non-amplified U-NB1 and SH-EP-MYCN-ER NB cells. In all three cell lines GSI-I induced a predominant G2/M arrest (Figure 4A, Supplementary Figure S7A and Figure 5B), in line with increased protein levels of p21, p27, CYCLIN B1, CDC25C, phospho-CDC25C Ser216 and SURVIVIN (Figure 4B). Protein levels of p16 and CDK4, which play a role in the G1 checkpoint, were also increased. GSI-I-treated cells exhibited signs of mitotic dysfunction, as shown by abnormal mitotic spindles in both SK-N-BE(2)C and U-NB1 NB cells (Figure 4C and Supplementary Figure S7B). Interestingly, mitotic dysfunction was not increased in either SK-N-BE(2)C or U-NB1 cells following treatment with the GSI DAPT (Figure 4C and Supplementary. Figure S7B).

Pro-apoptotic NOXA increased in GSI-I-treated SK-N-BE(2)C cells (Figure 4D and Supplementary Figure S8) and SH-EP-MYCN-ER cells (Supplementary Figure S8), consistent with induction of apoptosis that in part depends on caspases (Figure 4E). These changes in response to GSI-I were p53-independent, as the SK-N-BE(2)C cells investigated do not have functional p53 [41]. They also occurred irrespective of *MYCN* copy number, as SK-N-BE(2)C NB cells are *MYCN*-amplified, whereas SH-EP-MYCN-ER cells are not.

### Acute MYCN activation sensitizes NB cells to GSI-I

Next, we investigated whether GSI-I is efficacious in NB with acutely activated MYCN. To this end, SH-EP-MYCN-ER cells were used [39]. Activation of MYCN with 4-OHT was verified by strong increase of mRNA expression of *ODC1,* a target gene of MYCN (Figure 5A). Acute activation of MYCN forced SH-EP-MYCN-ER cells out of G0/G1 into S phase, leading to enhanced GSI-I-induced arrest in G2/M (Figure 5B), followed by increased apoptosis (Figure 5C). Thus, acute activation of MYCN sensitizes SH-EP-MYCN-ER NB cells to GSI-I.

Since we had shown above that GSI-I increases proapoptotic NOXA expression in SK-N-BE(2)C cells, we asked whether MYCN synergizes with GSI-I by also increasing NOXA. In SH-EP-MYCN-ER cells GSI-I treatment increased *NOXA* mRNA expression, as expected (Supplementary Figure S8). While *NOXA* mRNA expression increased upon acute activation of MYCN in the SH-EP-MYCN-ER cells, this increase did not reach significance (Supplementary Figure S8). In contrast, *in silico* analysis of 651 patient NB samples showed that *MYCN*-amplified patient tumors have significantly higher transcript levels of *NOXA* (Figure 5D).

Taken together, MYCN enhances GSI-I-induced G2/M arrest in SH-EP-MYCN-ER NB cells and is associated with increased transcription of *NOXA* in patient tumors. The latter may synergize with GSI-I-induced NOXA expression to induce apoptosis.

### GSI-I inhibits growth of a *MYCN* non-amplified orthotopic NB xenotransplant

**Figure 6:**
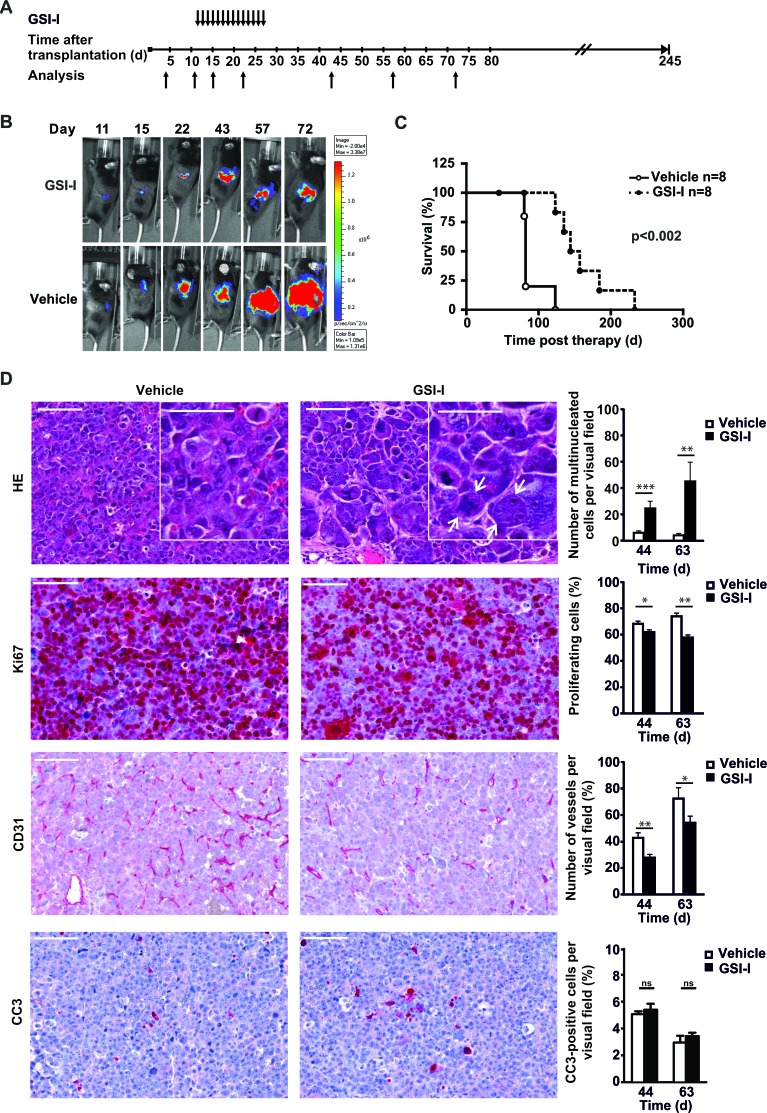
Systemic GSI-I decreases growth of NB xenografts and is associated with mitotic catastrophe, decreased proliferation and reduced tumor angiogenesis A Treatment schedule of orthotopically transplanted U-NB1 tumors. 2 × 10^5^ U-NB1 cells expressing luciferase were transplanted into the left adrenal gland of immunodeficient mice. Growth of tumors was monitored by luminescence imaging starting 4 days post transplantation. Treatment was started when tumors were detected in two consecutive measurements. Mice in control and treatment groups (n=8 for each group) were paired for similar tumor size and treated with 5 mg/kg GSI-I or vehicle only by daily i.p. injections for 14 days. **B**. GSI-I reduces growth of orthotopic U-NB1 xenotransplants. Shown is the course of one representative pair of tumors. For imaging, mice were injected with luciferin. **C**. Prolonged survival of NB-bearing mice treated with GSI-I. Shown is a Kaplan-Meier survival analysis. Mice were killed when they became moribund. Statistical analysis was performed by log rank test. **D**. GSI-I-treated NB show signs of mitotic catastrophe, decreased proliferation and reduced vascularisation compared to vehicle control. Mice received GSI-I (n=5) or vehicle only (n=4) 5 days a week by i.p. injections for 4 weeks. At different time points, one mouse per group was sacrificed. Tumor tissue sections were stained by HE, for Ki67, for CD31 and for cleaved caspase 3 (CC3). Shown are the results for 12 and 31 days after therapy (i.e. 44 and 63 days after tumor cell transplantation). Bars equal 100 μm and 50 μm (inserts). Statistical analysis was performed by unpaired one-sided t-test; *p<0.05, **p<0.01, ***p<0.001. Arrows point to giant multinucleated tumor cells.

To investigate whether *MYCN* non-amplified orthotopic NB xenotransplants are sensitive to GSI-I *in vivo* we transplanted U-NB1-luc NB cells [32] into the adrenals of immunodeficient mice. Mice were systemically treated with GSI-I or vehicle control (Figure 6A). GSI-I inhibited growth of tumors while not inducing regression (Figure 6B), translating into significantly prolonged survival (Figure 6C), without evidence of gastrointestinal toxicity (Supplementary Figure S9). Next, we analyzed the cellular effects of GSI-I treatment in U-NB1 tumors. To this end mice were treated for a longer period to increase treatment intensity. GSI-I-treated U-NB1 tumors were characterized by an increased number of aberrant mitotic figures and giant multinucleated cells typical for mitotic catastrophe (Figure 6D, upper row). GSI-I modestly decreased proliferation with multinucleated cells being mostly quiescent (Figure 6D, second row). Compared to vehicle-treated tumors the number of CD31-positive vessels was reduced in GSI-I-treated tumors (Figure 6D, third row). GSI-I-treated samples exhibited few cells with cleaved caspase 3 (Figure 6D, lower row).

Collectively, these data show that GSI-I blunted growth of orthotopic *MYCN* non*-*amplified NB cell tumors, associated with decreased proliferation, evidence of mitotic catastrophe and inhibition of tumor angiogenesis.

## DISCUSSION

In this study we have delineated that NOTCH is relevant for maintaining NB cells and that it constitutes a therapeutic target susceptible to inhibition by GSI-I. In addition to inhibiting NOTCH GSI-I interferes with the proteasome and possibly with additional cellular functions, thus mounting a multipronged attack on NB cells. To avoid undue generalization, it should be kept in mind that for reasons of feasibility only a limited number of NB cell lines was investigated in most of the experiments.

NOTCH was constitutively active in NB cells, as proven by NOTCH receptor cleavage and expression of NOTCH target genes. As NB cells express NOTCH ligands as well as receptors, both auto- and paracrine ligand-receptor interactions could be the cause of NOTCH activation.

Having confirmed that NOTCH is active in NB cells we interfered with NOTCH signaling. We chose γ-secretase as a target, as cleavage of NOTCH receptors is the common activator of NOTCH signaling and because little was known about the effects of inhibiting this enzyme in NB. Previous studies investigated the *in vitro* effect of the GSIs compound E and DAPT on two NB cell lines [12] and the short-term response of a subcutaneous NB cell line xenograft to a GSI called Jia142 [6]. Using a large panel of NB cell lines and low-passage NB cultures, we determined that GSI-I, not investigated previously in NB, was the most effective of several classes of GSIs investigated. We then showed *in vitro* that inhibition of γ-secretase by GSI-I blocks NOTCH signaling, since cleavage of NOTCH receptors, NOTCH reporter gene activity and expression of several NOTCH target genes all decreased upon treatment. Using a low-passage orthotopic xenograft we proved that GSI-I reduces NB growth and prolongs mouse survival. NOTCH inhibition correlated with cell toxicity suggesting that NOTCH inhibition is a relevant mechanism of GSI-I in controlling growth of NB cells.

Forced expression of dnMAML allowed us to compare the efficacy of GSI-I with specific, non-pharmacological inhibition of NOTCH signaling in NB cells. dnMAML decreased clonogenic growth, supporting a role of NOTCH in maintaining the malignant phenotype of NB cells. In SK-N-BE(2)C cells GSI-I treatment decreased colony formation more than did dnMAML, suggesting other mechanisms in addition to NOTCH inhibition that mediate cytotoxicity of GSI-I. Indeed, we observed that GSI-I also inhibits the proteasome in NB cells. Inhibition of the proteasome by GSI-I was weaker and, in the case of U-NB1, more transitory compared to the strong and sustained inhibition by MG132, a *bona fide* proteasome inhibitor structurally similar to GSI-I. Thus, part of the NB-controlling effect of GSI-I may be attributable to its proteasome-inhibiting effect. This most likely enhances efficacy of GSI-I against NB. While we have demonstrated that GSI-I inhibits the proteasome in NB, as it does in other cancers [21–26], the degree of inhibition was limited. This suggests that additional mechanisms of GSI-I are operational. Along this line, it remains to be investigated whether cleavage of other known substrates of γ-secretase by GSI-I [16–20] contributes to its efficacy in NB cells. As with other small molecules, yet undescribed off-target effects that may contribute to efficacy cannot be ruled out.

GSI-I caused G2/M arrest and mitotic dysfunction in NB cells, associated with up-regulation of molecules important for this cell cycle checkpoint, i.e. CYCLIN B1, CDC25C, pCDC25C Ser216 and SURVIVIN. Of note, proteasome inhibition has been described to arrest NB cells in G2/M, associated with increased CYCLIN B1 levels [29]. Thus, GSI-I-mediated cell cycle arrest in NB cells may be caused by its proteasome-inhibiting function leading to deregulated turnover of cell cycle regulators such as cyclins and CDKs. Inhibition of the proteasome leading to deregulation of CDC25C, p27 and cyclins has been shown to render cells more susceptible to apoptosis [28].

GSI caused marked mitotic dysfunction in SK-N-BE(2)C and U-NB1 NB cells *in vitro* and mitotic catastrophe in U-NB1 tumors *in vivo*. Interestingly, *in vitro* treatment with the GSI DAPT, thought to be specific for NOTCH, did not cause mitotic dysfunction. This suggests that the mitotic dysfunction and catastrophe induced by GSI-I is not caused by the inhibitory effect of GSI-I on NOTCH but rather by inhibiting proteasome function. Along this line, mitotic dysfunction has been shown to be induced by the proteasome inhibitor bortezomib in NB [42–44]. Other mechanisms yet to be determined may also be involved.

We have shown that GSI-I-treated NB cells undergo apoptosis, associated with increased pro-apoptotic NOXA. This may have been caused by decreased turnover of NOXA protein, in line with decreased protein turnover described in NB cells in response to proteasome inhibitors like bortezomib [45].

Systemic GSI-I inhibited growth of orthotopic NB xenografts. Regression of tumors was not noted. This suggests that inhibition of mitosis by GSI predominates over its pro-apoptotic effects. In addition, route, dosage and schedule of GSI-I administration may have been suboptimal. Thus, combining GSI-I with other drugs, and determining and improving pharmacokinetics of GSI-I warrant further investigations.

*In vitro*, NB cell lines with amplification of *MYCN* were, on average, as susceptible to GSI-I as NB cells with non-amplified *MYCN.* SH-EP MYCN-ER cells with acutely activated MYCN were even more sensitive to GSI-I compared to cells without activated MYCN. Activation of MYCN shifted cells from G0/G1 into S phase. This led to a markedly increased proportion of cells being arrested in G2/M phase by GSI-I. Thus, acute activation of MYCN enhances GSI-I-induced G2/M arrest. Taken together, even aggressive *MYCN*-amplified NB cells respond to GSI-I.

Along this line, MYCN and GSI-I cooperated to induce apoptosis in NB cells. It is conceivable that this cooperation is mediated by proapoptotic NOXA. Thus, GSI-I induced transcription of *NOXA* and increased its protein levels. The latter may have been caused by the proteasome inhibition induced by GSI-I, as proteasome inhibition preceded the increase of NOXA protein. A similar sequence of events has been described for GSI-I in chronic lymphocytic leukemia [26]. While *NOXA* mRNA expression increased upon acute activation of MYCN in the SH-EP-MYCN-ER cells, this increase did not reach significant levels (Supplementary Figure S8). In contrast, *in silico* analysis showed that *MYCN*-amplified patient tumors have significantly higher transcript levels of *NOXA* (Figure 5D), in line with recent data showing that MYCN increases *NOXA* expression in NB [46]. Taken together, while consistent with the notion of cooperation between MYCN and GSI-I in NB by mutual induction of NOXA, our data do not yet prove this notion.

We have provided evidence that GSI-I inhibits angiogenesis in NB, which may contribute to GSI-I-mediated tumor control *in vivo.* While GSI-I-mediated anti-angiogenesis has not been described yet, it is known that inhibition of NOTCH by other GSIs decreases tumor angiogenesis by blocking NOTCH signaling [47, 48]. Inhibition of the proteasome may play a role in the anti-angiogenic effect of GSI-I, as proteasome inhibitors have been reported to decrease tumor vasculature [28].

Of note, no intestinal metaplasia or weight loss was noted in mice treated with GSI-I. This contrasts with many other GSIs, where intestinal metaplasia constitutes a frequent and severe side effect. In addition to the lack of gastrointestinal toxicity, no other toxicities have been reported for GSI-I [49].

In conclusion, GSI-I with its activity on NOTCH signaling, proteasome activity and possibly additional cellular functions is effective against NB cells, without gastrointestinal toxicity in mice. Pharmacokinetic optimization and combination with other drugs may enhance its efficacy. Thus, further investigations of GSI-I for NB are warranted.

## MATERIALS AND METHODS

### Cell culture and cell lines

The human NB cell lines SH-SY5Y, IMR-32, GI-ME-N and Kelly were purchased from DSMZ (Braunschweig, Germany) and NB69 cells from the ECACC (Sigma-Aldrich, Munich, Germany). SK-N-BE(2)C, SK-N-SH and SK-N-AS NB cell lines were acquired from ATCC (LGC Standard, Wesel, Germany). AMC711T and U-NB1 NB cells have been described previously [31, 32]. U-NB2 cells were established from a 6 year-old patient with a stage IV, non-differentiated, retroperitoneal NB with amplified *MYCN*, tetrasomy of chromosome 2 and imbalance of 1p36. Generation of U-NB2 cells, culture conditions and authentication of NB cells are described in more detail in Supplementary Material and Methods.

### Chemicals

GSI-I, GSI-X, DAPT and compound E (Calbiochem, Darmstadt, Germany) were dissolved in dimethyl sulphoxide (DMSO, Sigma-Aldrich). Inhibitors were used at final concentrations of 100 nM to 20 μM, the final concentration of the solvent DMSO did not exceed 0.1% (vol/vol).

### Metabolic activity assay

MTT (Sigma-Aldrich) metabolic activity assays were performed as described [33]. Results were calculated relative to untreated controls from at least 4 wells per experimental condition.

### Soft agar assay

NB single cell suspensions of 1000 cells/ml were seeded in soft agar in 24-well plates. Growth medium with 1 μM GSI-I was replaced twice a week until colonies became visible.

### Sphere formation assay

NB cells were seeded in clonal density (1 cell/μl) into non-adhesive dishes in serum-free medium. EGF and bFGF were added twice a week.

### Western blot analysis

Cells were lysed in Laemmli lysis buffer with fresh protease and phosphatase inhibitors (Roche) [34]. Western blotting was performed as described in [34] using antibodies listed in Supplementary Information.

### Reverse transcription PCR analysis

Total RNA was isolated using TRIzol reagent and reverse transcribed by SuperScript III First-Strand Synthesis System according to the manufacturer's instructions (Invitrogen). Taq polymerase (Invitrogen), appropriate primers (Supplementary Tab. S1) and 28 cycles were used for amplification. Samples were separated on agarose gel stained with PeqGREEN (Peqlab, Erlangen, Germany). mRNA expression levels were determined relative to *ACTIN* expression.

### Luciferase assay

NB cells were transiently co-transfected with the expression plasmids pcDNA3-mNOTCH1-ΔE and pGL3-12*RBP-Jκ-Luc [35, 36] using Lipofectamine (Invitrogen). Luciferase activity of GSI-I-treated cells was determined from cleared cell lysates in a microplate reader.

### Proteasome assay

Proteasome assay was performed as described in [37]. Briefly, NB cells were treated with GSI-I (1 μM) or MG132 (0.75 μM, Calbiochem). 50 μg protein from cell lysate supernatants and 150 μM of Suc-LLVY-AMC (Bachem, Bubendorf, Switzerland) were incubated and fluorescence determined using the Mithras LB 940 microplate reader (Berthold) with excitation at 390 nm and emission at 460 nm.

### Cell cycle analysis

2 × 10^5^ NB cells were seeded in 6-well plates and treated with 1.5 μM GSI-I. Cells were harvested every 4-6 hours after treatment and fixed with 80% ethanol. DNA was stained with propidium iodide (PI, 40 μg/ml, Sigma-Aldrich) containing RNAse (100 μg/ml, Thermo Scientific). Cell cycle distribution of cells was analysed using flow cytometry.

### Histology, immunocytochemistry and immunohistochemistry

Histology and immunohistochemistry of formalin-fixed, paraffin-embedded sections, and immunocytochemistry of formalin-fixed NB cell lines were performed using standard protocols with antibodies listed in Supplementary Information.

For quantifying mitotic dysfunction using immunocytochemistry aberrant mitotic figures were counted in 3 slides per experiment from 3 independent experiments and were calculated as percentage of total mitotic figures.

For analysis of mitotic catastrophe in tissue slides mitotic catastrophe was defined as cells with micronuclei or multiple nuclei. 3 visual fields at 20 x magnification in 3 slides per tumor from 2 tumors were analyzed.

For analysis of proliferating tumor cells, tumor vessels, and apoptotic tumor cells in tissue slides 3 visual fields at 20 x magnification in three slides per tumor from three tumors were analyzed. Representative fields were chosen, i.e. non-necrotic homogeneous areas with many proliferative tumor cells or vessels, so-called hotspots. For quantifying proliferating cells those positive for Ki67 were counted, for tumor vessels linear structures of CD31-positive endothelial cells were enumerated and for apoptotic cells those positive for cleaved caspase 3 (CC3) were analyzed.

### Apoptosis assay

Specific apoptosis was assessed by enumerating propidium iodide-stained hypodiploid nuclei by flow cytometry as described [38]. The percentage of specific apoptosis was calculated as follows: (experimental apoptosis (%) - spontaneous apoptosis in medium (%)) / (100% - spontaneous apoptosis in medium (%)) x 100.

### MYCN-ER translocation

SH-EP cells expressing MYCN-ER [39] were cultured with and without 300 nM 4-hydroxytamoxifen (4-OHT, Sigma-Aldrich). The MYCN target gene *ODC1* was assessed by quantitative RT-PCR using LightCycler Fast Start DNA Master SYBR green I (Roche, Basel, Switzerland). Primer sequences are listed in Supplementary Table S1. Relative expression levels were calculated using the 2^−ΔΔCt^ method and were normalized to the reference gene Hypoxanthine phosphoribosyltransferase 1 (*HPRT1)*.

### Correlation between *NOXA* mRNA expression and *MYCN* amplification status of NB patient samples

Clinically annotated mRNA expression profiles generated from 651 primary NB patients using a 44k oligonucleotide array were used [40].

### Orthotopic NB mouse model and *in vivo* therapy

For the orthotopic xenotransplant model viable *MYCN* non-amplified U-NB1-luc cells in 30 μl of 25% high concentration matrigel^TM^ (BD Biosciences, Heidelberg, Germany) were surgically implanted into adrenal glands of 6-8 week old female RAG2^−/−^/cγc^−/−^ mice. Treatment was started when tumors became visible by bioluminescence imaging (Xenogen, IVIS 200, Caliper life sciences, Mainz, Germany), corresponding to a tumor volume of 20 μl, as determined in pilot studies (data not shown). Mice were treated with 5 mg/kg/day GSI-I dissolved in 6% DMSO/30% CremophorEL/PBS (Sigma) or vehicle by daily i.p. injection. Mice were sacrificed when moribund.

All experiments were performed according to institutional and state guidelines for the care and protection of animals.

### Statistical analysis of *in vitro* and *in vivo* experiments

GraphPad Prism 6.01 software (La Jolla, CA, USA) was used.

Additional information is provided in Supplementary Material and Methods.

## SUPPLEMENTARY MATERIAL FIGURES AND TABLES


