# Supplementary material for: γ-secretase inhibitor I inhibits neuroblastoma cells, with NOTCH and the proteasome among its targets
Source: Oncotarget. 2016 Aug 30;7(39):62799–813. doi: 10.18632/oncotarget.11715 (PMC5325329; doi:10.18632/oncotarget.11715)
Supplement: Supplementary file 1 [file oncotarget-07-62799-s001.pdf]

γ-secretase inhibitor I inhibits neuroblastoma cells, with NOTCH and the proteasome among its targets

Supplementary Information

Methods

Cell culture and cell lines

IMR-32, Kelly, SH-EP, LAN5, NB69, GI-ME-N and U-NB1 cells were cultured in RPMI 1640 (GIBCO, Paisley, UK) with 10% fetal bovine serum (FBS, GIBCO), 2 mM glutamine (GIBCO) and 100 U/ml penicillin/streptomycin (GIBCO). SK-N-AS, SH-SY5Y and SK-N-SH cells were cultured in DMEM (GIBCO) with 10% FBS, 2 mM glutamine and 100 U/ml penicillin/streptomycin. SK-N-BE(2)C cells were cultivated in EMEM and Ham's F12 (1:1) with 10% FBS, 2 mM glutamine, 100 U/ml penicillin/streptomycin and 1 x non-essential amino acids (all from GIBCO). AMC711T cells were cultured in DMEM:Ham's F12 (3:1) (GIBCO) supplemented with 2% B27 (GIBCO), EGF (20 ng/ml, Miltenyi Biotech, Bergisch Gladbach, Germany), FGF (40 ng/ml, Miltenyi Biotech) and 100 U/ml penicillin/streptomycin. For controls, DMSO was added to the medium in a concentration of 0.1% corresponding to the final concentration of DMSO used in the experimental groups. Final concentration of DMSO never exceeded 0.1% (vol/vol). LAN5 and U-NB1 cells were grown on collagen-coated cell culture material. All cell lines were incubated at 37°C in a humidified atmosphere with 5% CO_2.

Authentication of NB cells

The human cell lines were obtained from DSMZ or ATCC in July 2012, April 2014 and most recently in June 2015, and have been authenticated by short tandem

repeat (STR) profiles. For STR profiling of cell lines the GenePrint 10 System (Promega, Mannheim, Germany) was used according to the manufacturer's recommendation. For STR profiling of short-term cultures forensic STR loci were determined by the Institute of Forensics, Ulm University.

Generation and genetic characterization of U-NB2 cells

Tumor tissue was cut and dissociated with liberase DH (0.62 U/ml in HBSS, Roche, Mannheim, Germany) at 37°C for 15-45 min. Cells were resuspended in serum-free DMEM/F12 medium (GIBCO) supplemented with 2 mM glutamine, 100 U/ml Pen/Strep, 10 IU/ml heparin (ratiopharm, Ulm, Germany), 20 ng/ml LIF (Millipore, Billerica, MA, USA), 20 ng/ml basic FGF (Miltenyi Biotech), 20 ng/ml EGF (Miltenyi Biotech) and 1x B27 (50x, GIBCO) seeded into non-adhesive cell culture dishes and passaged weekly. EGF and basic FGF were added to the tumor cells every 23 days.

To detect chromosomal aberrations, we performed fluorescence in situ hybridization (FISH). U-NB2 cells were treated with pepsin (0.05 mg/ml) for 3 min at 37°C. For fixation samples were incubated 5 min in 1% PFA/PBS, washed with PBS and dehydrated with increasing ethanol concentrations. Samples were denatured with denaturation mix 70% (v/v) deionized formamide, 2x SSC (20x SSC: 3 M NaCl, 0.3 M sodium citrate pH 7), 7 ml NaPO4 buffer (pH 7) at 72°C for 2 min. After dehydration and drying the probes were added. Probes were from Abbott Molecular (Wiesbaden, Germany). To detect 1p36 microdeletion the Locus Specific identification probe (LSI) p58 (1p36) orange/TelVysion 1p green/LSI 1q25 aqua was used. For *MYCN* amplification the Vysis LSI N-MYC (2p24) green/chromosome enumeration probe (CEP) 2 (2p11.1-q11.1) orange was used. Probe mix (7 µl hybridization buffer, 1 µl DNA probe, 2 µl dH2O) was denaturated at 73°C for 5 min. The probe mix was added onto the sample, covered with a cover slip, sealed with rubber cement and

incubated at 37°C overnight. Slides were washed with buffer A (50% formamide, 2x SSC, pH 7-7.2) at 42°C and in buffer B (0.2x SSC pH 7-7.2) at 60°C. Whole DNA was stained with DAPI (200 ng/ml in 2x SSC) for 5 min and washed with buffer D (2x SSC, 0.1% Tween 20) for 3 min. Finally, samples were mounted with fluorescent mounting medium and analyzed with a fluorescence microscope.

CD11a, CD19 and CD20 flow cytometry

U-NB1 and U-NB2 cells were stained for the lymphocyte markers CD11a, CD19 and CD20. $2x10^5$ cells per sample were used for staining. PBMCs isolated from buffy coat served as positive staining controls. CD11a was detected using anti-human CD11a-FITC mouse IgG1 (eBiosciences). Cells were incubated at room temperature in 3% BSA/PBS containing 4 µl CD11a antibody or unspecific FITC-conjugated IgG1 isotype control (BD Pharmingen) for 30 min. For staining of CD19 mouse anti-human CD19-FITC IgG1 (Beckman Coulter) was used. To detect CD20 the samples were treated with anti-human CD20-FITC mouse IgG3 (Caltec) or appropriate isotype control mouse IgG3-FITC (BD Pharmingen). Finally, samples were washed twice to remove unbound secondary antibody and analysed by flow cytometry (FACS Calibur, Beckman Coulter).

Immunocytochemistry

Formalin-fixed NB cells were probed with mouse-anti-alpha-TUBULIN (Calbiochem), mouse-anti-Peripherin (Sigma-Aldrich) or rabbit-anti-Chromogranin A (Origene) and secondary anti-mouse or anti-rabbit IgG Alexa Fluor 488 and Hoechst 33258 (Invitrogen), and were analysed by fluorescence microscopy using the LSM 710 laser scanning microscope and Zen10 software (Carl Zeiss MicroImaging, Jena, Germany) and a Keyence BZ-9000 microscope (Neu-Isenburg, Germany).

Immunohistochemistry

Immunohistochemistry of formalin-fixed, paraffin-embedded sections was performed using standard protocols. Briefly, serial sections were deparaffinized, pressure-cooked in citrate buffer, treated with avidin/biotin blocking reagent (DAKO, Hamburg, Germany), and probed with anti-CD31 (Dianova, Hamburg Germany), rabbit-anti-Tyrosine hydroxylase (Pel-Freez, Rogers, USA) or rabbit-anti-human-active caspase 3 (1:250, Abcam). Alkaline phosphatase-conjugated anti-mouse or anti-rabbit and liquid permanent red substrate chromogen system (DAKO) were used according to the manufacturers' instructions. Ki67 (DAKO) staining was performed using the EnVision G/2 System/AP rabbit/mouse permanent Red protocol (DAKO). Stained samples were analyzed using a Keyence BZ-9000 microscope.

Soft agar assay

0.6% soft agar was prepared using low melting point agarose (Invitrogen, Carlsbad, CA, USA) in growth medium containing FBS and additives. A single cell suspension of 1000 cells/ml in 0.3% top agar was added onto the bottom agar in 24-well plates. Growth medium with 1 µM GSI-I was replaced twice a week until colony formation was observed. Colonies were stained with 1 mg/ml MTT (Sigma).

Clonogenic growth assay

A single cell suspension of NB cells in clonal density, i.e. 1000 cells/well was seeded in 6-well plates. Growth medium with 1 µM GSI-I was added 24h after seeding. Colonies were stained with crystal violet (Sigma-Aldrich) after 5-7 days.

Sphere formation assay

NB cells were seeded in clonal density (1 cell/µl) into non-adhesive dishes in serum-free DMEM/F12 medium (GIBCO) supplemented with 2 mM glutamine, 100 U/ml penicillin/streptomycin, 10 I.U./ml heparin (ratiopharm, Ulm, Germany), 20 ng/ml LIF (Millipore, Billerica, MA, USA), 20 ng/ml basic FGF (Miltenyi Biotech, Bergisch Gladbach, Germany), 20 ng/ml EGF (Miltenyi) and 1x B27 (GIBCO). EGF and bFGF were added twice a week.

Western blot analysis

Cells were lysed in Laemmli lysis buffer with fresh protease and phosphatase inhibitors (Roche). Primary antibodies against cleaved-NOTCH2 (Val1697, Sigma-Aldrich), CYCLIN D1, CDK4, CYCLIN B1, CDK1 and CHK1 (Santa Cruz Biotechnology, Santa Cruz, CA, USA), cleaved-NOTCH1 (Val1744), CDC25C and pCDC25C Ser216 (Cell Signaling, Denver, MA, USA), pCDK1 Tyr15 (Biomol, Hamburg, Germany), RB, SURVIVIN, p21, p16 and p27 (BD Pharmingen, Heidelberg, Germany), pRB (Abcam, Cambridge, UK), NOXA (Enzo Life Sciences, Lörrach, Germany) and GAPDH (HyTest, Turku, Finland) were applied over night. Goat-anti-mouse-HRP and goat-anti-rabbit-HRP (Santa Cruz) were used as secondary antibodies.

Generation and transfection of pcDNA3-dnMAML-GFP

The dnMAML-GFP cDNA was isolated by BglII/ClaI digestion of the retroviral vector pMIGR1-MAML(12-74)-GFP (1). After blunt end generation by Klenow the fragment was ligated into the EcoRV site of pcDNA3 (Invitrogen) to generate pcDNA3-dnMAML-GFP. 4 x 10^5 NB cells were transiently transfected with lipofectamine 2000 (Invitrogen) in 6-well plates with 5 µg pcDNA3-dnMAML-GFP.

Luciferase assay

NB cells (4 x 10^5) were transfected with lipofectamine 2000 (Invitrogen) in 6-well plates with 5 µg of RBP-Jκ-Luc reporter plasmid together with 5 µg mNOTCH1-ΔE expression plasmid. GSI-I treatment was started 24h post transfection and Luciferase activity was determined from at least three independent transfections with 20 µl of cleared cell lysates at indicated time points in a Mithras LB940 microplate reader (Berthold, Bad Wildbad, Germany) using the Luciferase Assay System (Promega, Mannheim, Germany).

PAS-staining

Periodic acid-Schiff (PAS) staining was performed according to standard protocols. Briefly, formalin-fixed, paraffin-embedded tissue specimens were deparaffinized and rehydrated. Periodic acid (Carl Roth, Karlsruhe, Germany) was added for 5 min and slides were incubated at room temperature. After washing in distilled water Schiff's reagent (Carl Roth) was added for 15 min at room temperature. Subsequently, specimens were washed in running tap water for 5 min and nuclei were counterstained with Meyer's hematoxylin (Sigma-Aldrich) for 5-10 sec. Finally, samples were dehydrated and mounted in non-aqueous mounting medium (Merck, Darmstadt, Germany).

Supplementary information bibliography

1. Maillard I, Weng AP, Carpenter AC, Rodriguez CG, Sai H, Xu L, Allman D, Aster JC, Pear WS. Mastermind critically regulates Notch-mediated lymphoid cell fate decisions. Blood. 2004;104:1696-702.

Supplementary Fig. S1

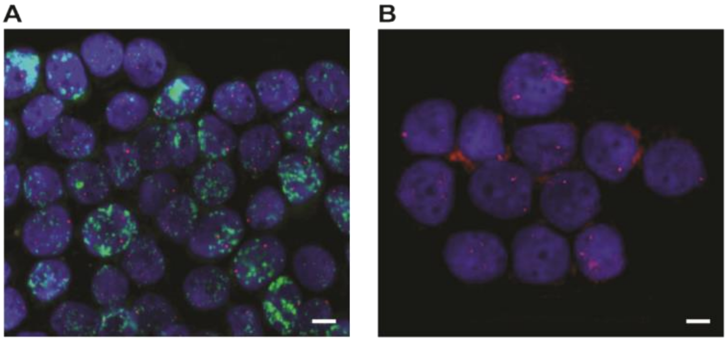

Supplementary Fig. S1 U-NB2 cells are *MYCN* amplified and not 1p36 deleted U-NB2 sphere cells derived from orthotopically xenotransplanted tumors were analyzed for chromosomal aberrations by FISH.

(A) *MYCN* is highly amplified in U-NB2 cells. DNA probes against *MYCN* (2p24) (green) and CEP (2p11.1-q11.1) (red) were used.

(B) 1p36 is not deleted in U-NB2 cells. For detection of 1p36 a DNA probe against p58 (red) was used.

Bars equal 5 µm.

Supplementary Fig. S2

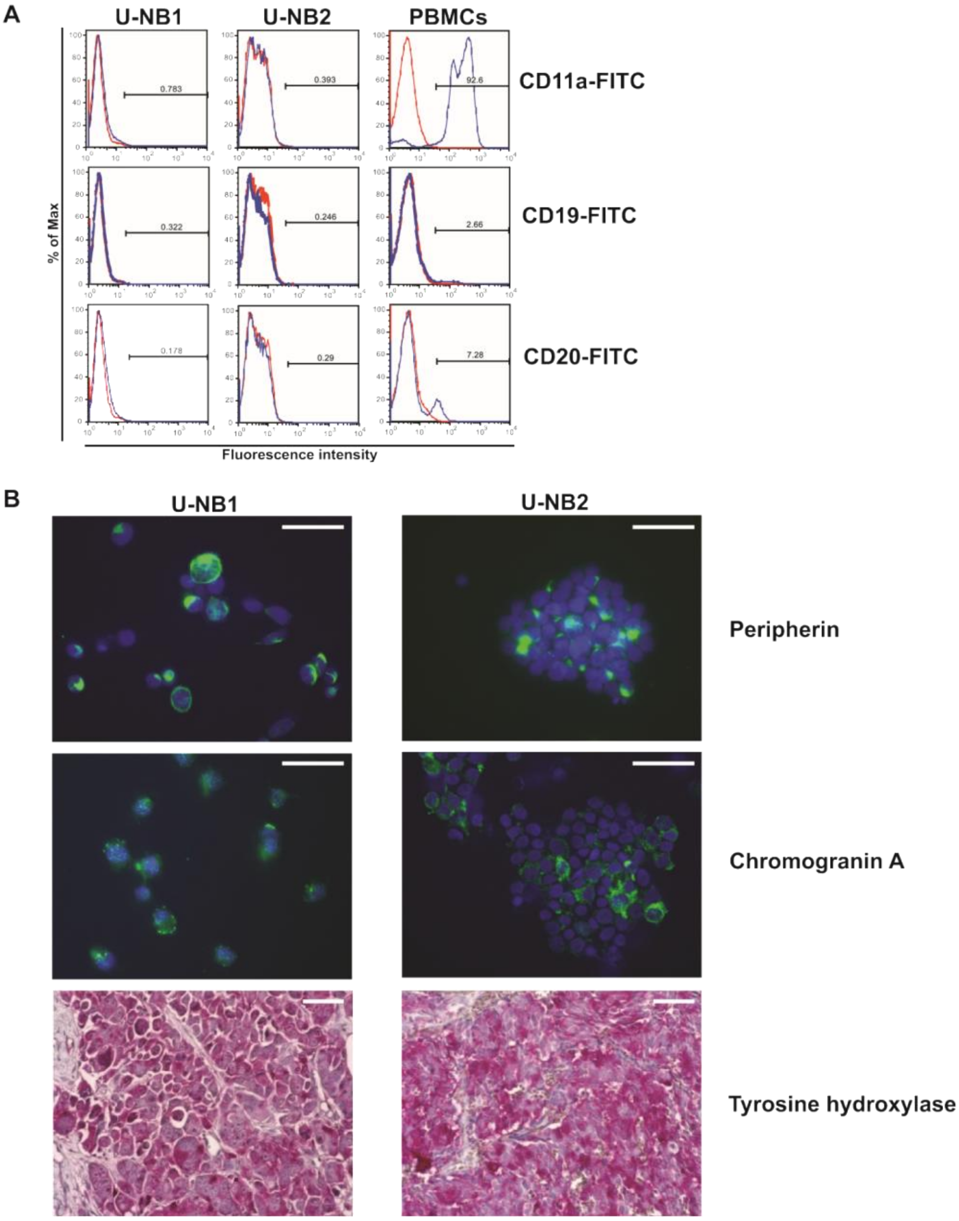

Supplementary Fig. S2 U-NB1 and U-NB2 primary short-term cultures do not express lymphocyte markers but express NB-associated markers

(A) U-NB1 and U-NB2 are negative for CD11a, CD19 and CD20. U-NB1, U-NB2 and PBMCs (as positive control) were stained for CD11a, CD19 and CD20, and analysed by flow cytometry. Histograms show positively stained cell fractions (blue lines) and isotype controls (red lines).

(B) U-NB1 and U-NB2 cells express peripherin, chromogranin A and tyrosine hydroxylase. U-NB1 cells grown on cover slips and U-NB2 cytospin samples were fixed with 4% formalin and were stained for peripherin (green) and chromogranin A (green). Nuclei were stained with Hoechst. Formalin-fixed, paraffin-embedded tissue samples of U-NB1 and U-NB2 xenotranplants were stained for tyrosine hydroxylase. Bars equal 50 µm. Experiments were repeated three times, with similar results.

Supplementary Fig. S3

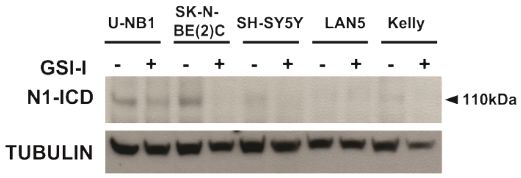

Supplementary Fig. S3: The NOTCH1 intracellular domain (N1-ICD), detectable at low levels in some NB cell lines, is decreased by GSI-I. NB cell lines were treated with GSI-I for 48 h. Levels of N1-ICD were determined by Western blot analysis. Tubulin was used as loading control.

Supplementary Fig. S4

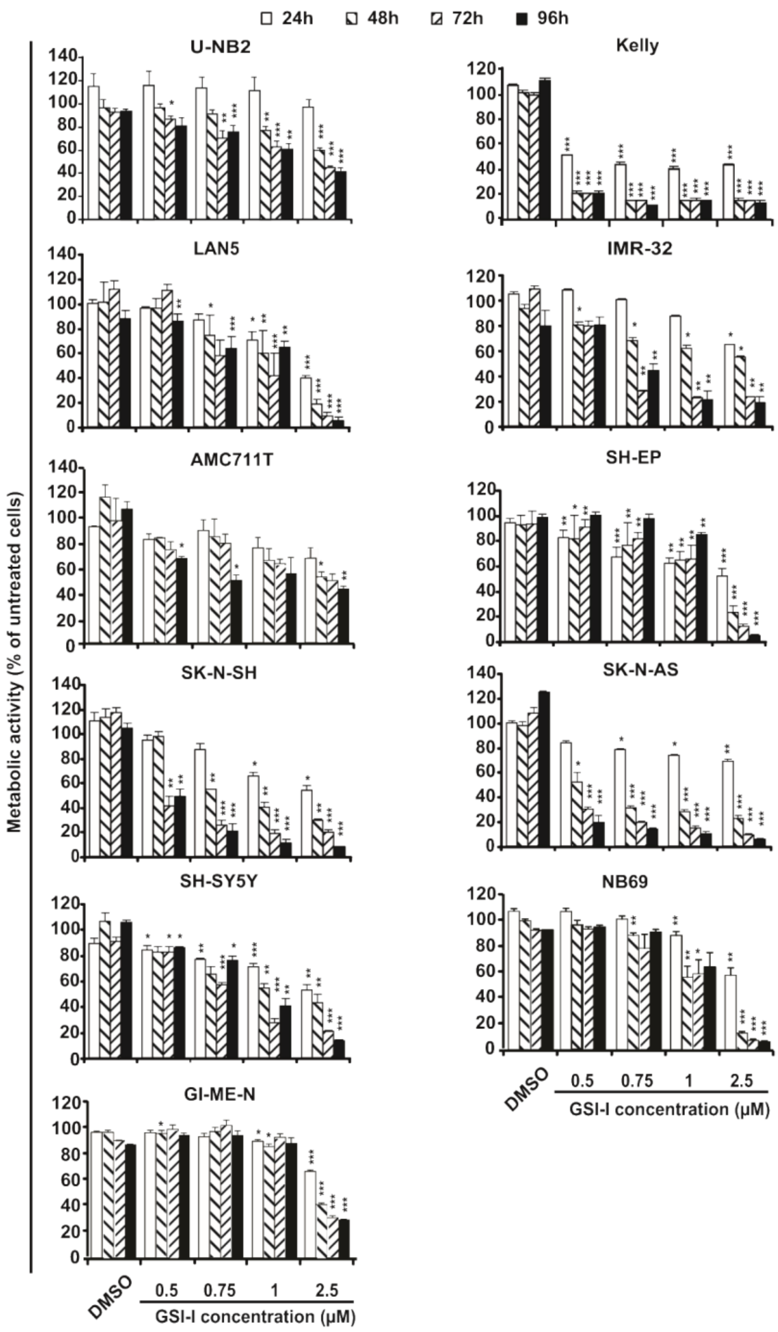

Supplementary Fig. S4 GSI-I reduces metabolic activity of NB cells in a dose- and time-dependent manner. NB cell lines were seeded in 96-well plates and treated with GSI-I. Metabolic activity was determined by MTT assay. Means and standard deviations were calculated from quadruplicates. Statistical significance was evaluated using the unpaired t-test. *p<0.05, **p<0.01, ***p<0.001. Experiments were repeated three times, with similar results.

Supplementary Fig. S5

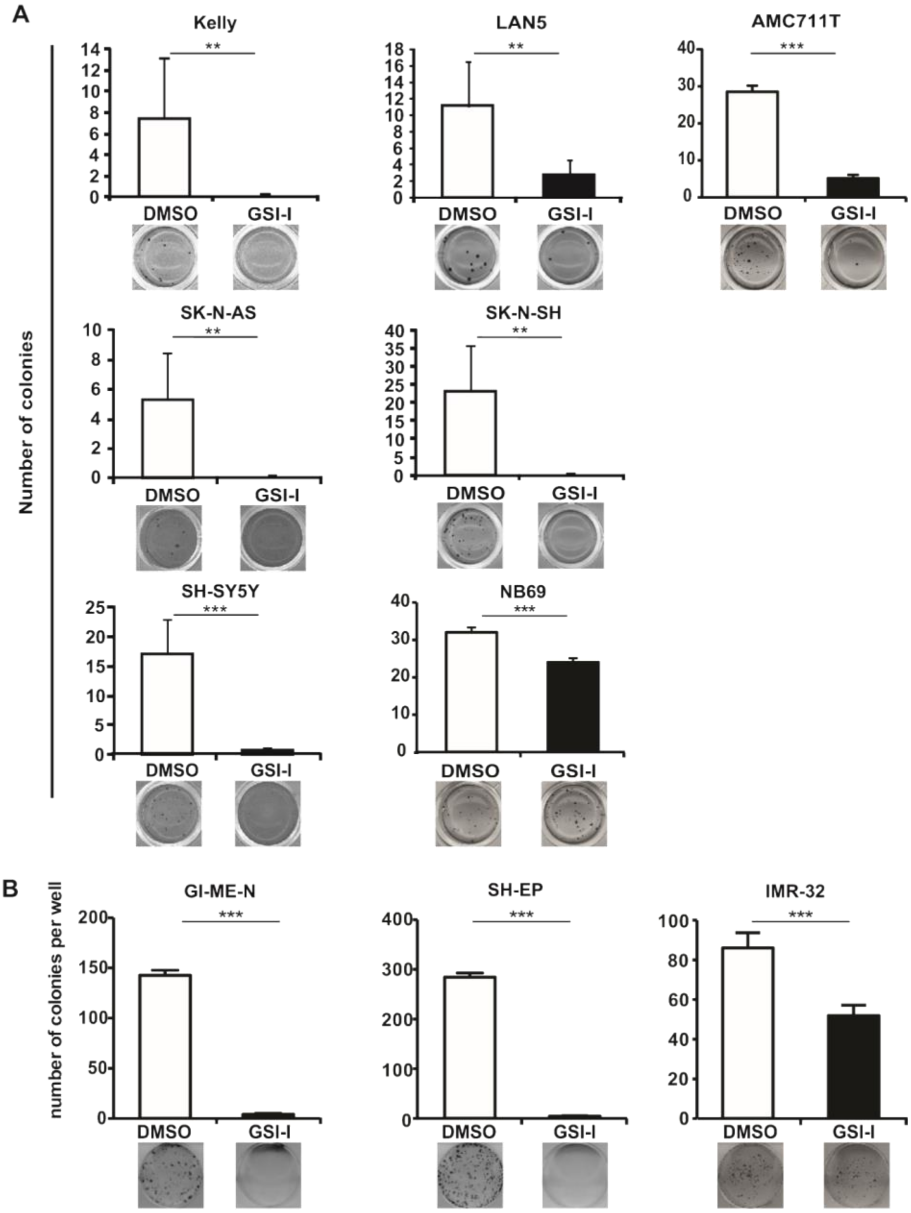

Supplementary Fig. S5 GSI-I markedly reduces clonogenicity of NB cells

(A) GSI-I markedly decreases anchorage-independent growth of NB cells. NB cell lines were seeded in clonal density, i.e. 1000 cells/ml in 24-well plates in soft agar. Cells were treated with 1 µM GSI-I. After 20-30 days colonies were stained with MTT. Means and standard deviations of 12 wells per experimental condition are shown. **p<0.01, ***p<0.001, using the unpaired t-test. Experiments were repeated at least three times, with similar results.

(B) GSI-I abolishes clonogenic growth of GI-ME-N, SH-EP and IMR-32 NB cells. As GI-ME-N, SHEP and IMR-32 cells do not grow in soft agar, clonogenic growth on plastic was assayed. NB cells were seeded at 1000 cells/well in 6-well plates. Cells were treated with 1 µM GSI-I. After 5-7 days colonies were stained with crystal violet. Means and standard deviations of 9 wells per experimental condition are shown. ***p<0.001, using the unpaired t-test.

Supplementary Fig. S6

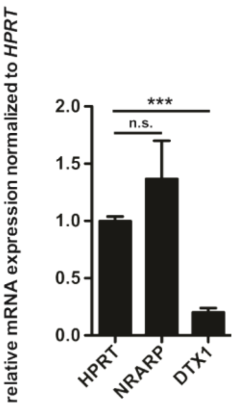

Supplementary Fig. S6 dnMAML reduces expression of the NOTCH target gene *DTX1*. SK-N-BE(2)C cells were transiently transfected with dnMAML-GFP or empty vector control. 96 h after transfection RNA was isolated and transcribed into cDNA. qRT-PCR of *NRARP* and *DTX1* was performed and analysed relative to *HPRT*. Means and SD from three independent experiments are shown. p values were calculated using the t-test; ***p<0.001; n.s. not significant.

Supplementary Fig. S7

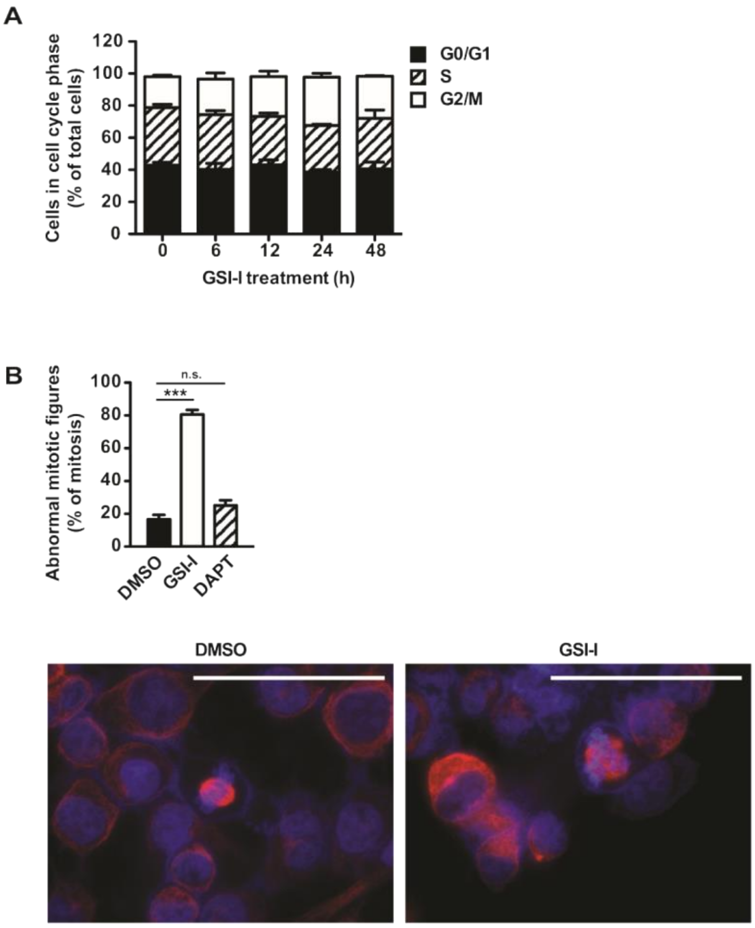

Supplementary Fig. S7 GSI-I affects mitosis in U-NB1 cells

(A) GSI-I induces G2/M cell cycle arrest. U-NB1 cells were treated with 1.5 µM GSI-I for the indicated times. Cells in G1, S and G2/M phases were determined by FACS analysis. Means and SD were calculated from three independent experiments.

(B) GSI-I induces mitotic dysfunction. U-NB1 cells were treated on cover slips with 1 µM GSI-I or DAPT for 24 h. Formalin-fixed samples were stained for α-tubulin (red) and DNA (blue). The graph shows the number of abnormal mitotic figures counted as the percentage of total mitotic figures. Means and SD were calculated from three independent experiments and the p value was determined using the t-test; ***$p<0.001$; n.s. not significant. In the lower panel representative pictures of cells treated with DMSO or GSI-I are shown. Bars equal 100 µm. Experiments were repeated three times, with similar results.

Supplementary Fig. S8

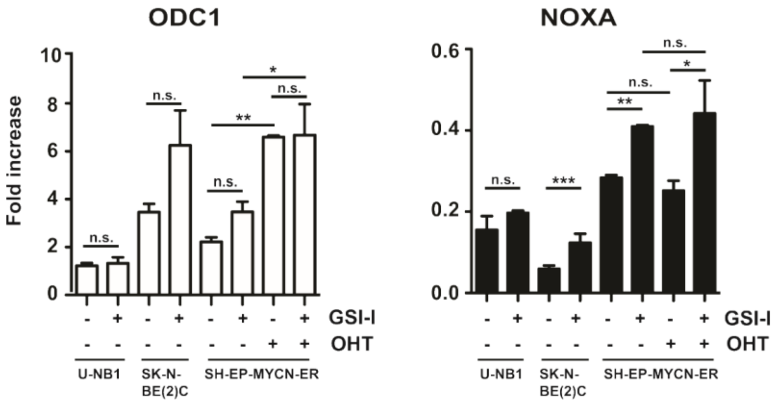

Supplementary Fig. S8 GSI-I but not acute activation of MYCN increases *NOXA* expression. U-NB1 and SK-N-BE(2)C cells were treated for 48 h with 1 µM (U-NB1) or 1.5 µM (SK-N-BE(2)C) GSI-I. SH-EP-MYCN-ER cells were treated with 300 µM 4-OHT and 1 µM GSI-I for 48 h. qRT-PCR of *ODC1* and *NOXA* mRNA was performed. mRNA expression was calculated relative to HPRT. Means and SD were calculated from independent qRT-PCR runs and p values were determined using the t-test; ***p<0.001, **p<0.01, *p<0.05, n.s. not significant.

Supplementary Fig. S9

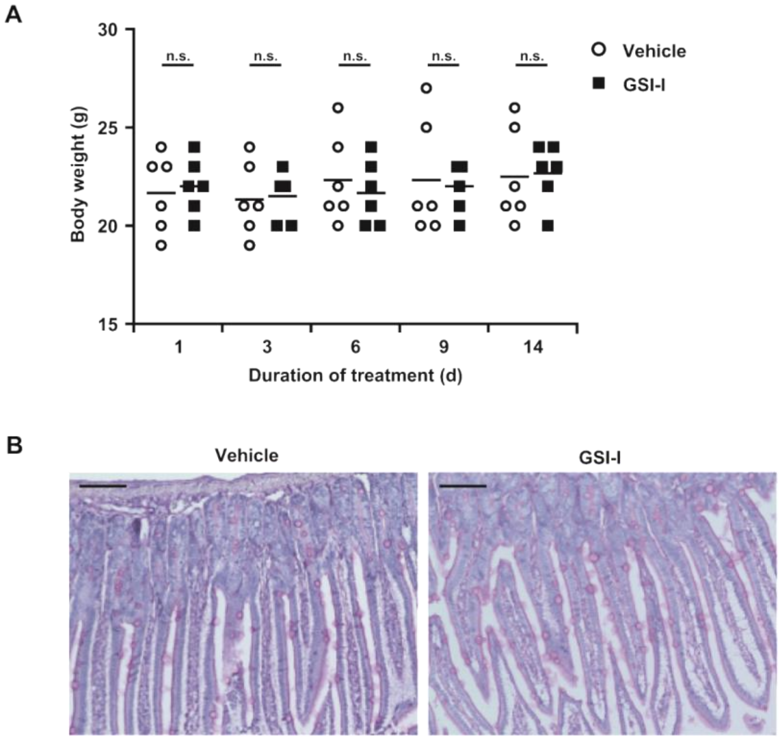

Supplementary Fig. S9 Lack of gastrointestinal toxicity in GSI-I-treated mice
(A) No weight loss induced by GSI-I. 2 x 10^5 U-NB1 cells stably expressing luciferase were injected into the left adrenal gland of immunodeficient mice. The formation of tumors was monitored by luminescence imaging starting 4 days post transplantation. Treatment was started when tumors were detected in two consecutive measurements. Mice in control and treatment groups were paired for similar tumor size and were treated with 5 mg/kg GSI-I or vehicle only by daily i.p. injections for 14 days. The weight of mice was measured regularly during treatment.
(B) GSI-I does not cause intestinal metaplasia. At the end of treatment the small intestine was procured and subjected to PAS-staining to detect goblet cells. Bars represent 100 µm.

Supplementary Table S1: Primer pairs used for RT-PCR analysis. Fwd, forward primer; Rev, reverse primer; [1]RT-PCR, [2]qRT-PCR.

Gene	Sequence	Annealing tem-perature (°C)	$MgCl_2$ (mM)
**NOTCH1**[1]	Fwd-CGGAGCTTCCTGAGTGGAGA<br>Rev-ACCAGTCGGAGACGTTGGAA	58	1.5
**NOTCH2**[1]	Fwd-GCCTGTGTCCTCCTGGTTTC<br>Rev-CTGGCAGTTGCACTGGTAGC	59	1.5
**NOTCH3**[1]	Fwd-ACTTTGGCCCTGCTCAAAAA<br>Rev-TCTTCTTGGACCCCGACTGT	55	1.5
**NOTCH4**[1]	Fwd-CATGATCACTTCCACAACGG<br>Rev-AAGGAAGCGGAGTAGAAGCC	63	1.5
**DLL1**[1]	Fwd-CTGATGACCTCGCAACAGAA<br>Rev-CAGATCGGCTCTGTGCAGTA	57	1.5
**DLL3**[1]	Fwd-TGAGCATGGCTTCTGTGAAC<br>Rev-TCAAAGGACCTGGGTGTCTC	58	1.5
**DLL4**[1]	Fwd-TGCAGGAGTTCATCAACGAG<br>Rev-ACATAGTGGCCGAAGTGGTC	58	1.5
**JAG1**[1]	Fwd-AGACATCGATGAATGTGCCA<br>Rev-CCACAGACGTTGGAGGAAAT	63	1.5
**JAG2**[1]	Fwd-GATACCACCCCGAATGAGGA<br>Rev-AGCTTCCTTGCACTCCTTGC	57	1.5
**HES1**[1,2]	Fwd-GGAGAGGCGGCTAAGGTGTT<br>Rev-CGCTGTTGCTGGTGTAGACG	57	1.5
**HES5**[1]	Fwd-AAGCACAGCAAAGCCTTCGTCGC<br>Rev-GCCTTCCGGAGAAGGGCGCG	65	2.0
**HES6**[1]	Fwd-AGCTCCTGAACCATCTGCTCG<br>Rev-GCCTGACTCAGTTCAGCCTCA	61	1.5
**HEY1**[1,2]	Fwd-CGAGGTGGAGAAGGAGAGTG<br>Rev-CTGGGTACCAGCCTTCTCAG	57	1.5
**NRARP**[1]	Fwd-GGCCACCATCCCCCAAACCG<br>Rev-GGCCCAGGTCTCGACGCAAC	63	1.5
**NRARP**[2]	Fwd-GGGCTGCATAGAAAATTGGA<br>Rev-CCCTTTTTAGCCTCCCAGAG	60	1.5
**DTX1**[1]	Fwd-ACTCCAATGGCAACAAGGAT<br>Rev-GGGGGATGAGGTGGAACT	61	1.5

**NOXA**[1,2]	Fwd-GCTGGAAGTCGAGTGTGCTA<br>Rev-CCTGAGCAGAAGAGTTTGGA	67	1.5
**ODC1**[2]	Fwd-GCCCGCTGTGTTTTTGACAT<br>Rev-ACGCCGGTGATCTCTTCAAA	60	2.5
**HPRT1**[2]	Fwd-CCTGGCGTCGTGATTAGTGA<br>Rev-CGAGCAAGACGTTCAGTCCT	60	2.5
**β-ACTIN**[1,2]	Fwd-TCACCCTGAAGTACCCCATC<br>Rev-TAGCACAGCCTGGATAGCAA	57	1.5
